# Supplementary material for: PARP2 mediates branched poly ADP-ribosylation in response to DNA damage
Source: Nat Commun. 2018 Aug 13;9:3233. doi: 10.1038/s41467-018-05588-5 (PMC6089979; doi:10.1038/s41467-018-05588-5)
Supplement: Supplementary file 1 — Supplementary Information [file 41467_2018_5588_MOESM1_ESM.pdf]

Supplementary Information

PARP2 mediates branched polyADP-ribosylation in response to DNA damage

Chen et al.

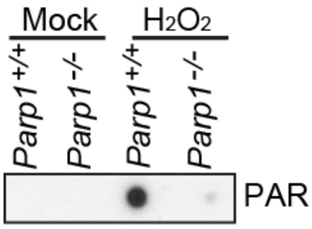

Supplementary Fig. 1. Loss of PARP1 largely impairs DNA damage-induced PARylation. *Parp1*$^{+/+}$ and *Parp1*$^{-/-}$ MEFs were treated with 500 μM H_2O_2. The levels of PAR were examined using dot blotting.

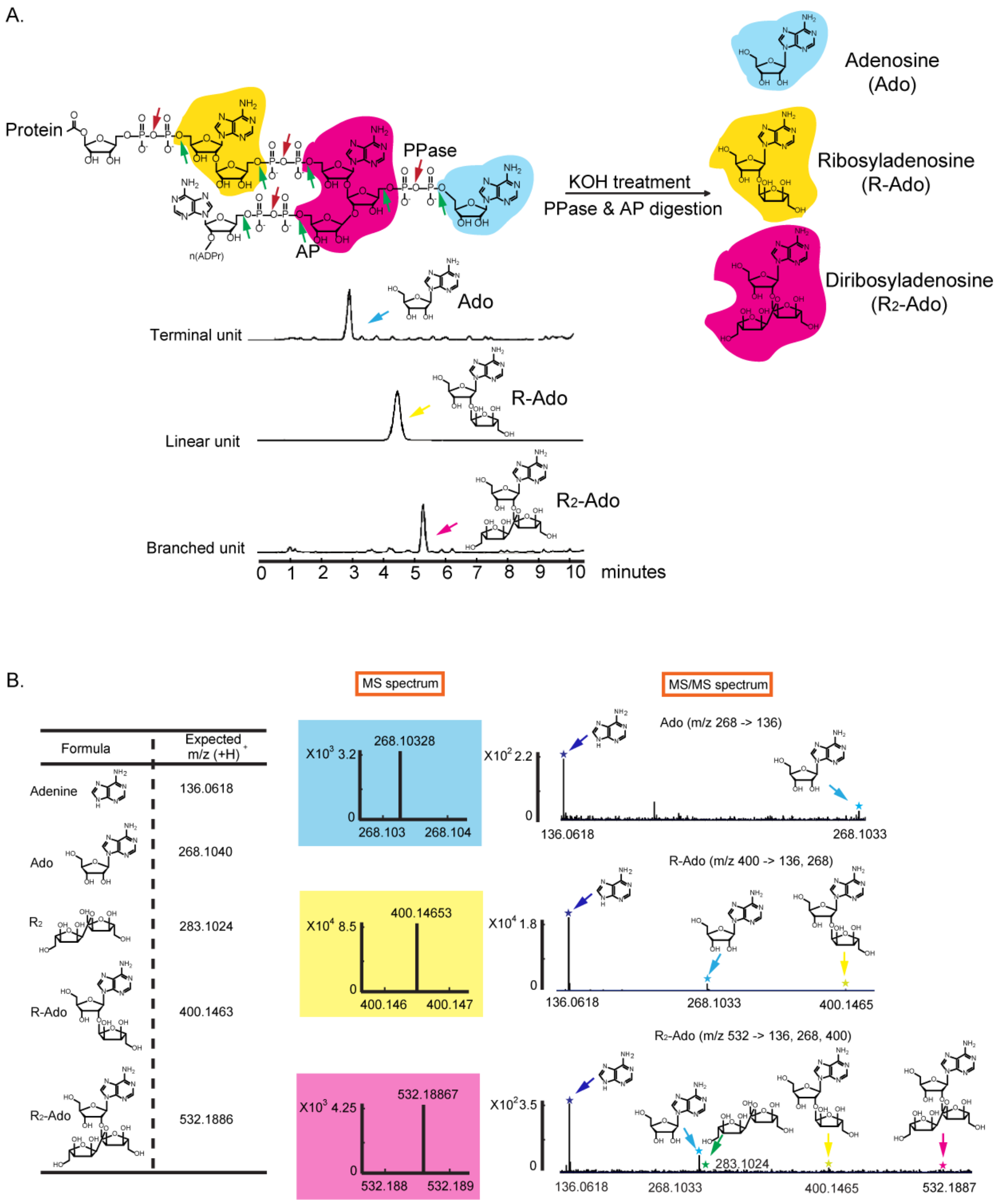

Supplementary Fig. 2. PAR is examined by LC-MS/MS. (A) Adenosine (Ado), ribosyladenosine (R-Ado) and diribosyladenosine (R_2-Ado) were obtained from PAR digestion by pyrophosphatase (PPase) and alkaline phosphatase (AP). Ado, R-Ado and R_2-Ado indicate the terminal, linear and branched PAR units, respectively. (B) Daughter ions scan of Ado, R-Ado and R_2-Ado were examined to confirm the parent ions. The expected m/z for individual molecule is indicated.

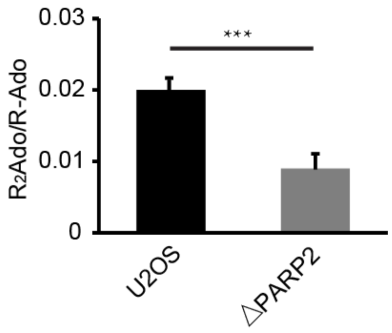

Supplementary Fig. 3. PARP2 is involved in branched PAR chain formation.

Depletion of PARP2 reduced the percentage of branched chain. The levels of R_2-Ado and R-Ado were measured by LC-MS/MS. The ratio of R_2-Ado/R-Ado suggests branch frequency. Data are represented as mean ± s.d. as indicated from three independent experiments. Significance of differences was evaluated by Student's t test. ***: Statistically Significant ($p < 0.001$).

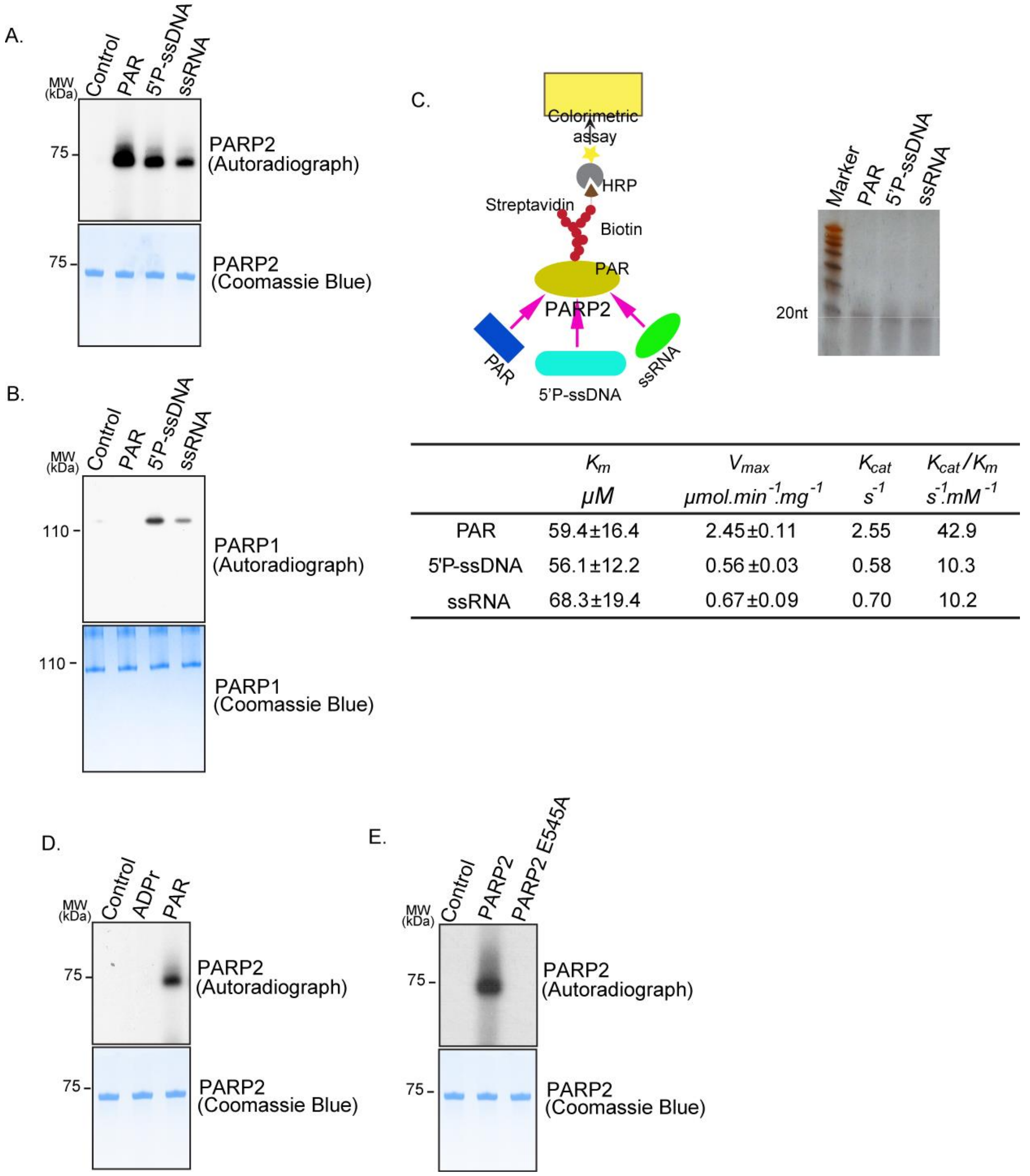

	K_m μM	V_{max} $\mu mol.min^{-1}.mg^{-1}$	K_{cat} s^{-1}	K_{cat}/K_m $s^{-1}.mM^{-1}$
PAR	59.4±16.4	2.45±0.11	2.55	42.9
5'P-ssDNA	56.1±12.2	0.56±0.03	0.58	10.3
ssRNA	68.3±19.4	0.67±0.09	0.70	10.2

Supplementary Fig. 4. PARP2 is activated by PAR. (A, B) *in-vitro* auto-PARylation of PARPs. Recombinant PARP2 (A) or PARP1 (B) incubated with ^{32}P-NAD$^+$ and various activators including PAR, 5'P-ssDNA or ssRNA. Auto-PARylation was detected by autoradiography (upper panel). Recombinant protein in each reaction was also examined by the SDS-PAGE with Coomassie blue staining (lower panel). (C)

Diagram representing work-flow for colorimetric assay. The *Kcat*/*Km* was measured using colorimetric assay. The level of PARP2 activators were examined by 20% native PAGE. (D) PARP2 cannot be activated by ADPr. (E) Only wide type PARP2, but not the E545A mutant is able to be activated by PAR.

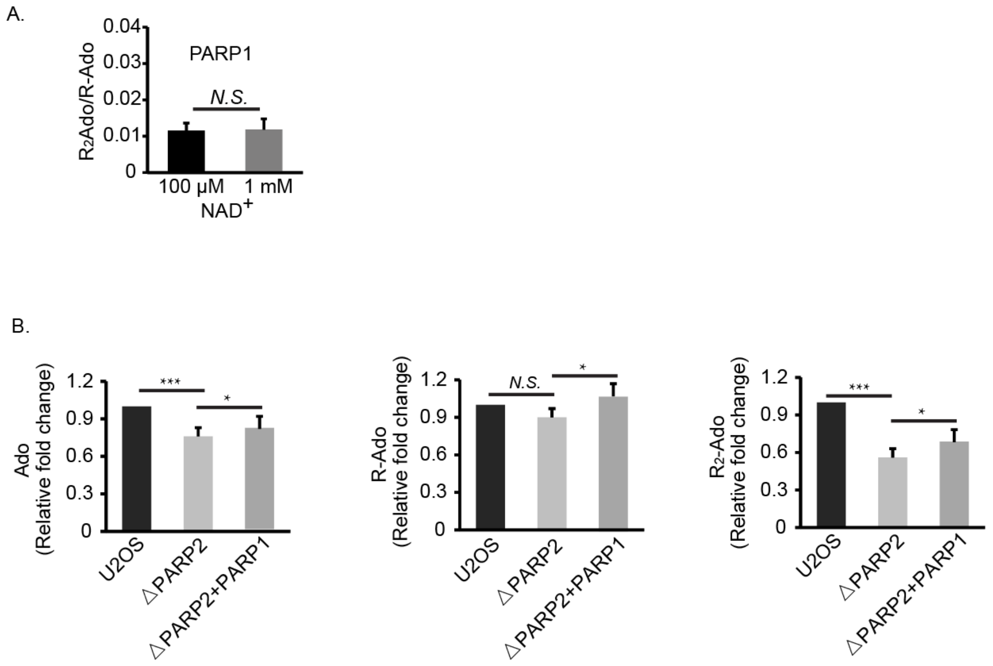

Supplementary Fig. 5. PARP1 is also able to catalyze branched chain, but at lower rate. (A) Measurement of branched chain in PARP1-dependent PARylation. The ratio between R_2-Ado and R-Ado was calculated. (B) Additional PARP1-dependent PARylation was analyzed in the PARP2 null cells over-expressing PARP1. Data are represented as mean ± s.d. as indicated from three independent experiments. Significance of differences was evaluated by Student’s t test. *N.S.*: Non Significant; *: Statistically Significant ($p < 0.05$). ***: Statistically Significant ($p < 0.001$).

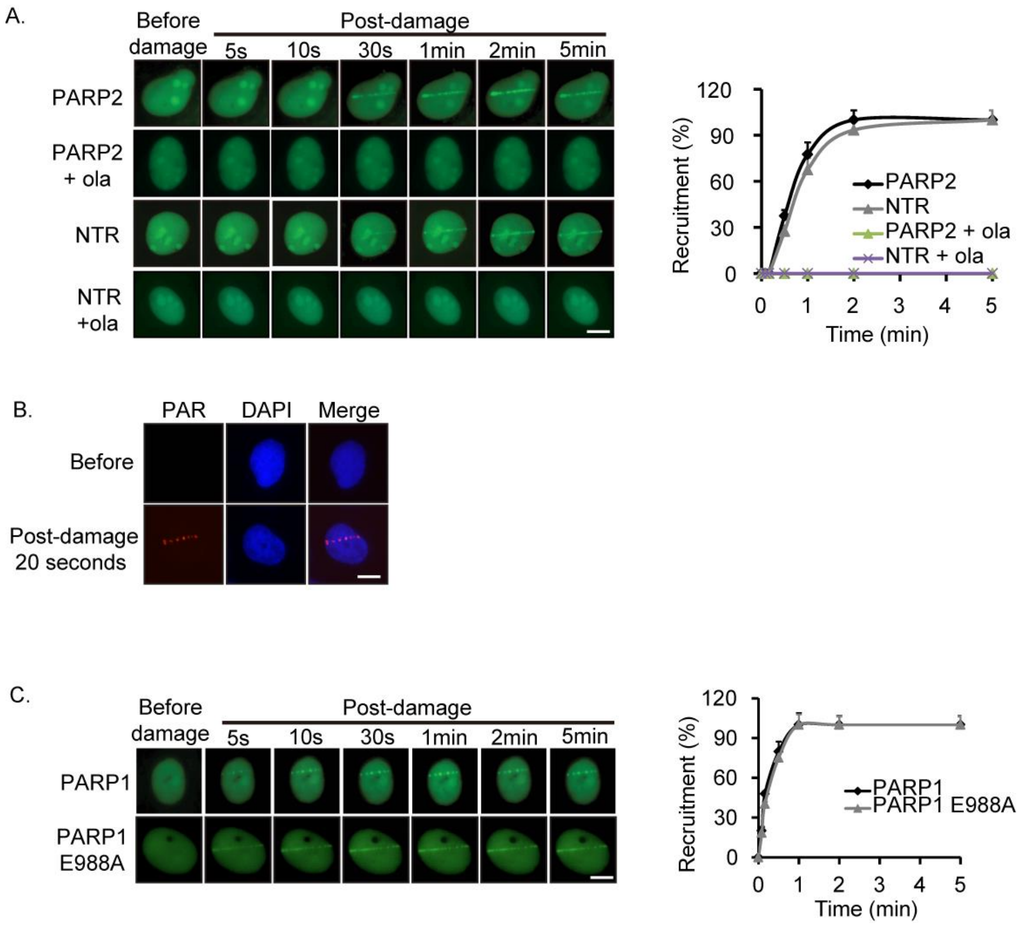

Supplementary Fig. 6. PARylation is essential for the PARP2 recruitment into the DNA damage sites. (A) Recruitment of either full-length PARP2 or NTR could be suppressed after PARylation inhibition. (B) PAR was detected at DNA lesions. PARylation was examined by immunostaining using anti-PAR antibody. (C) Catalytic inactive mutant of PARP1 (E988A) still could be recruited into the sites of DNA damage. Scale bar represents 5 μm.

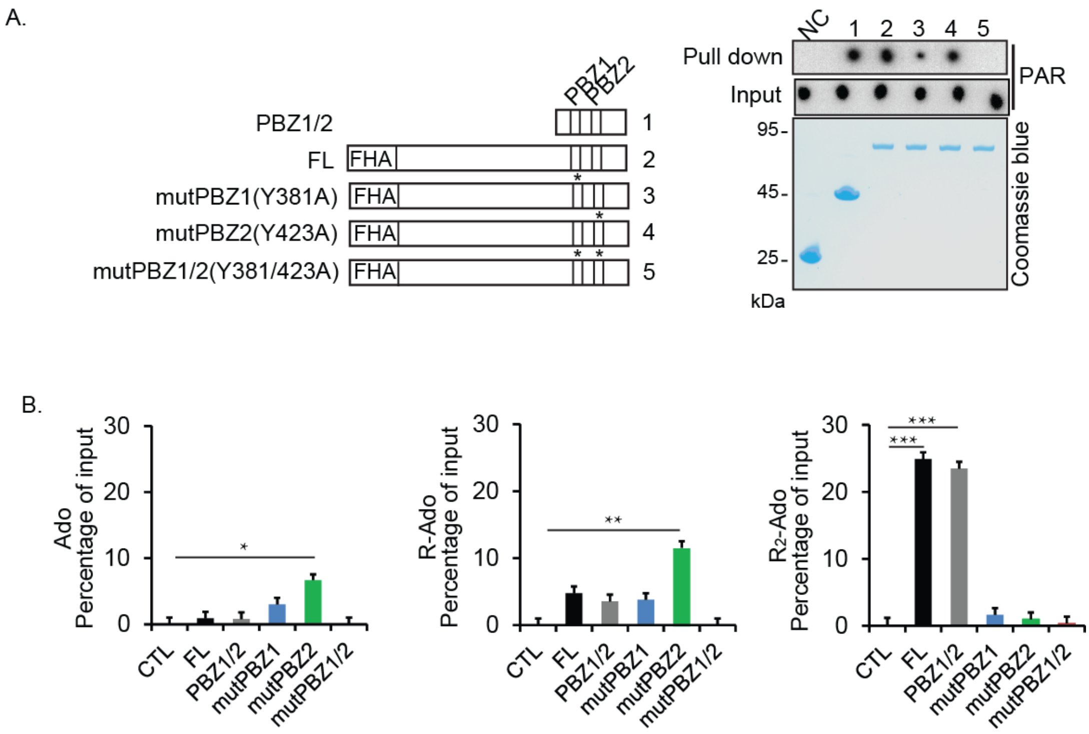

Supplementary Fig. 7. Loss of PBZ motifs abolishes the binding to the branched sites. (A) Mutation the key residue in each PBZ impairs the PAR-binding. The recombinant GST fusion proteins were incubated with ^{32}P labeled PAR. Protein-PAR complex was pulled down by glutathione agarose beads followed by autoradiography (left panel). Recombinant GST was used as the negative control (NC). The GST fusion proteins were also examined by the SDS-PAGE followed with Coomassie blue staining (right panel). (B) The pull down materials was further examined by mass spectrometry. Data are represented as mean ± s.d. as indicated from three independent experiments. Significance of differences was evaluated by Student's t test. *: Statistically Significant ($p < 0.05$). **: Statistically Significant ($p < 0.01$). ***: Statistically Significant ($p < 0.001$).

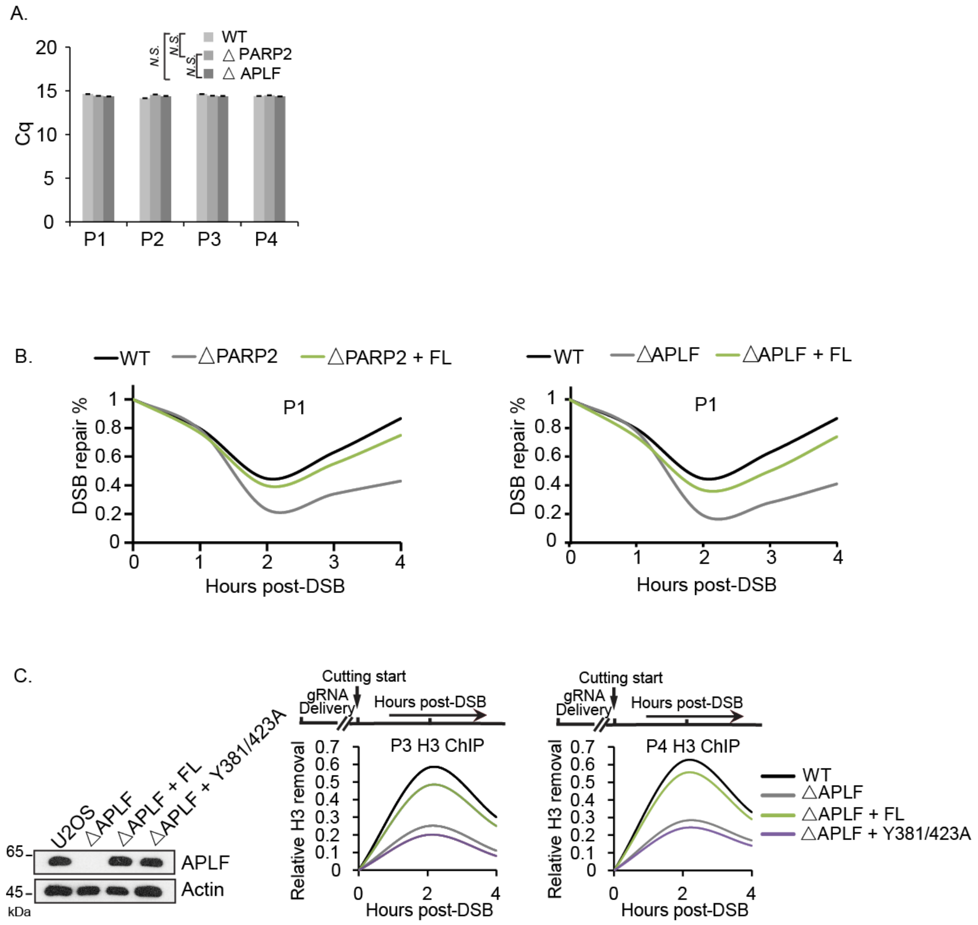

Supplementary Fig. 8. Both PARP2 and APLF are essential for DSB repair. (A) Absolute histone H3 levels at the four loci were examined by q-PCR in the PARP2- or APLF- deficient cells. (B) The DNA repair defect could be rescued by full length PARP2 or APLF (FL). (C) The PBZ motifs of APLF are important for the histone removal at the DSB. APLF-deficient cells (ΔAPLF) were reconstituted with full length APLF (FL) or the PBZ mutant (Y381/423A). The enrichments of histone H3 at DSB were examined by ChIP assays at indicated time points. Data are represented as mean ± s.d. as indicated from three independent experiments. Significance of differences was evaluated by Student's t test. *N.S.*: Non Significant.

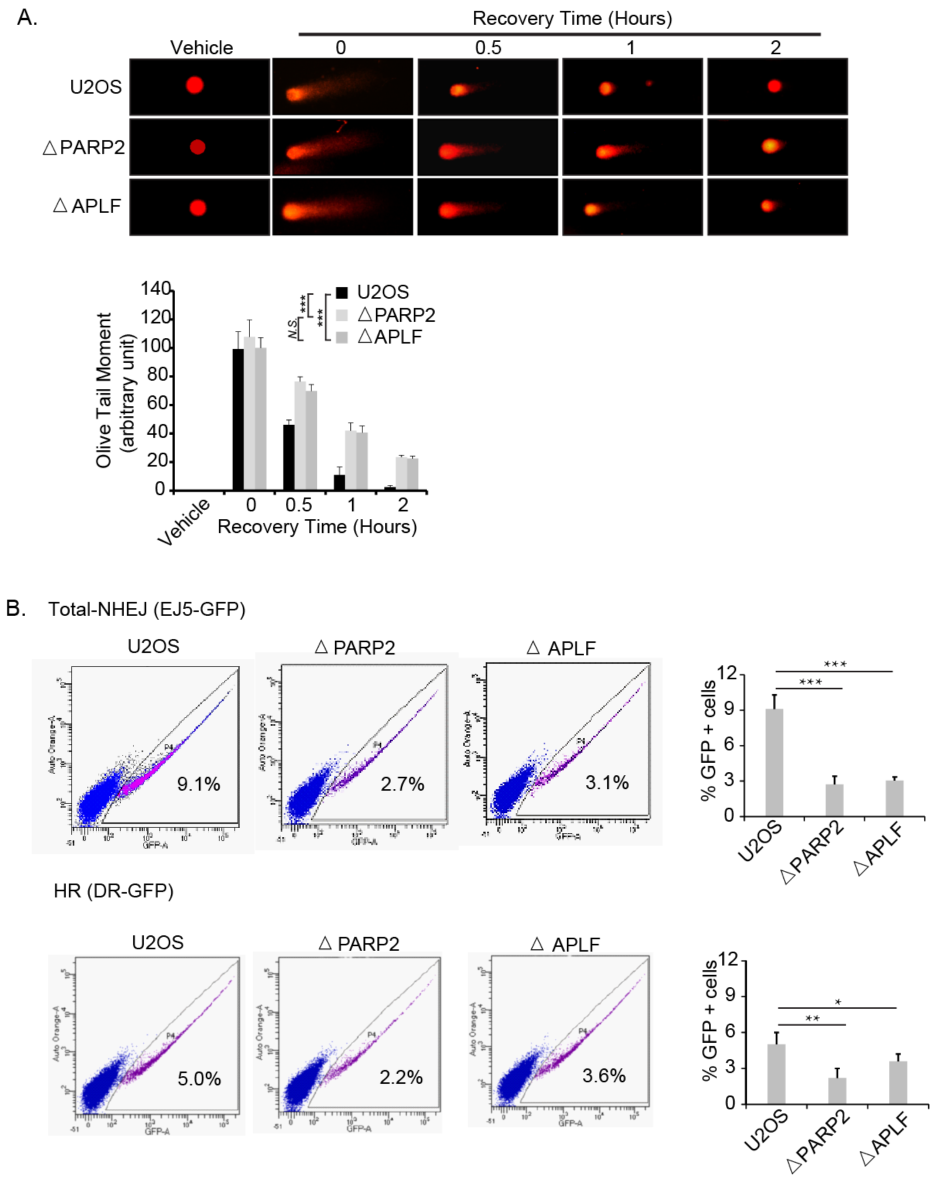

Supplementary Fig. 9. PARP2 and APLF play important roles in DSB repair. (A) The kinetics of DSB repair was examined with neutral comet assays in the PARP2- or APLF- deficient cells. Tail moments were measured from 50 cells and summarized in the histogram. (B) Both PARP2 and APLF participate in NHEJ (EJ5-GFP) or HR repair (DR-GFP). NHEJ or HR repair were examined in U2OS cell expressing the reporter GFP and lacking PARP2 or APLF. Data are represented as mean ± s.d. as indicated from three independent experiments. Significance of differences was

evaluated by Student’s t test. *N.S.*: Non Significant; *: Statistically Significant ($p < 0.05$). **: Statistically Significant ($p < 0.01$). ***: Statistically Significant ($p < 0.001$).

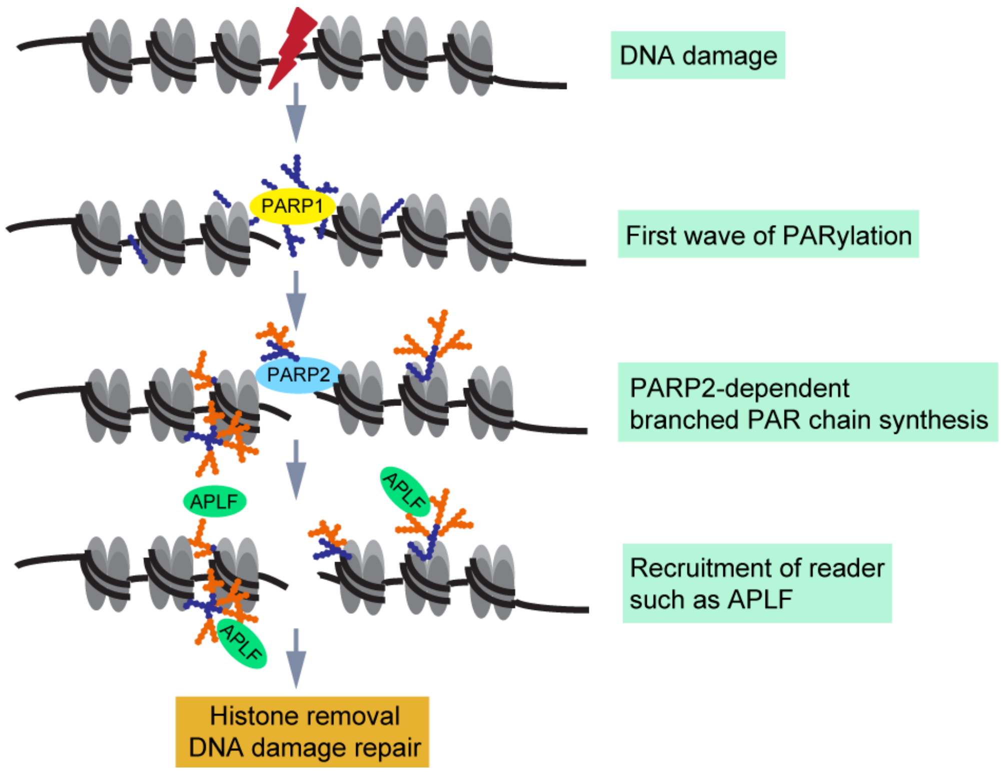

Supplementary Fig. 10. PARP2-dependent PARylation is a sequential step following initial PARP1-dependent PARylation during DNA damage repair.

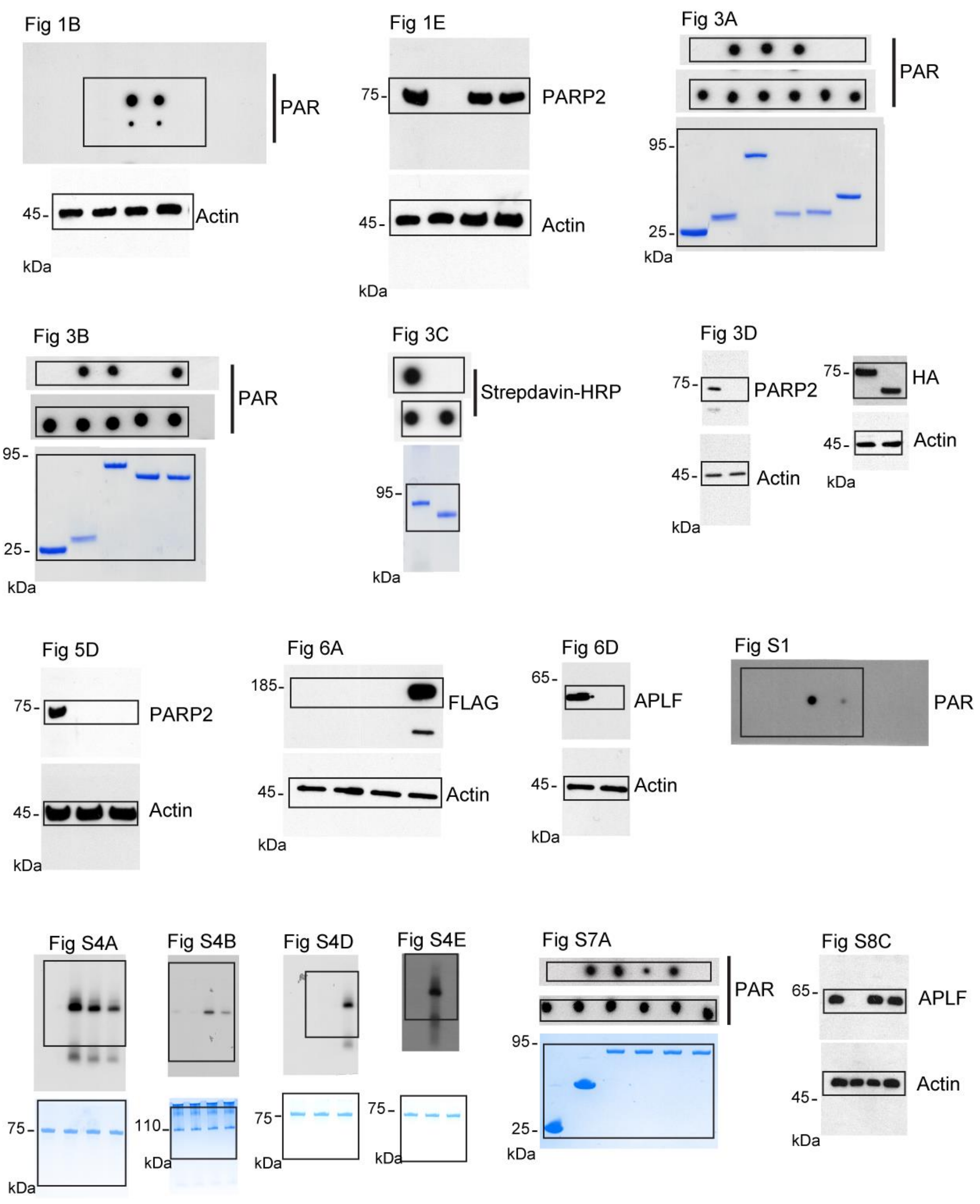

Supplementary Fig. 11. Original images of all blots and gels shown in this study.

Supplementary Table 1. List of primers used in PARP2 constructs

Primer Name	Sequence (5'-3')
FL PARP2 Forward	ATGGCGGCGCGGCGGCGA
FL PARP2 Reverse	TCACCACAGCTGAAGGAA
PARP2 NTR Forward	ATGGCGGCGCGGCGGCGA
PARP2 NTR Reverse	CTGGGCTGTACACTCTG
PARP2 WGR Forward	TCTGAATCTGTGAAG
PARP2 WGR Reverse	TTTCTTTGTTTCCTC
PARP2 CAT Forward	TATGATATGCTACAGATG
PARP2 CAT Reverse	TCACCACAGCTGAAGGAA
ΔNTR Forward	TCTGAATCTGTGAAG
ΔNTR Reverse	TCACCACAGCTGAAGGAA
ΔWGR Forward	GCCACCAATACTCAGGATGA
ΔWGR Reverse	AGCCTTCCCCACCTTGGCTGT
PARP2 E545A Forward	CTCAACTACAATGCATATATTGTATATAAC
PARP2 E545A Reverse	GGTATAACCATCTGGATTCAG

Supplementary Table 2. List of primers used in APLF constructs

Primer Name	Sequence (5'-3')
FL APLF Forward	ATGTCCGGGGGCTTCGAG
FL APLF Reverse	TATTTTCTTTTTCATAAAC
mutPBZ1(Y381A) Forward	CATCCTGCATGGCTGGGGCAAACTG
mutPBZ1(Y381A) Reverse	TCCTCTTGACCTTGTTTCC
mutPBZ1(Y423A) Forward	TGAATGTCCCGCTGGACCATCCTG
mutPBZ1(Y423A) Reverse	GGCCGGTCATCAGTCTCATC
APLF PBZ1/2 Forward	TCACTTCAGGATGAGTCTC
APLF PBZ1/2 Reverse	TATTTTCTTTTTCATAAAC

Supplementary Table 3. List of primers used in histone H3 removal examination

Primer Name	Sequence (5'-3')
P1 Forward	GTCACCAATCCTGTCCCTA
P1 Reverse	CAGGACAGCATGTTTGCTGC
P2 Forward	GGGAAATGGAGTCCATTAG
P2 Reverse	CCTGTAGACTCCATTTCCCA
P3 Forward	GCACCAGACGGCCGCGTCAG
P3 Reverse	CCAGAGAGGATCCTGGGAGG
P4 Forward	GAATCCACAGGAGAACGGG
P4 Reverse	CGTCCCGCCTCCCCTTCTTG
GAPDH Forward	GAGGGAGGTAGAGGGGTGAT
GAPDH Reverse	ATCACGCCACAGTTTCCCG

Supplementary Table 4. List of antibodies used in the study

Antibody	Company	Catalog #	Source	Application	Dilution
PARP2	Millipore	MABE18	Mouse monoclonal antibody	Western Blot	1:1000
APLF	Thermo Fisher Scientific	PA5-39776	Rabbit polyclonal antibody	Western Blot	1:1000
FLAG	Sigma	F3165	Mouse monoclonal antibody	Western Blot	1:5000
γH_2AX	Abcam	ab11174	Rabbit polyclonal antibody	Immunofluorescence	1:1000
Actin	Sigma	A2228	Mouse monoclonal antibody	Western Blot	1:5000
histone H3	Millipore	06-755	Rabbit polyclonal antibody	ChIP	1:500
PAR	Trevigen	4335-MC-100	Mouse monoclonal antibody	Dot Blot<br>Immunofluorescence	1:2000<br>1:1000
HRP-Streptavidin	Thermo Fisher Scientific	N100		Dot Blot	1:2000
